# HeH^+^ under Spatial Confinement

**DOI:** 10.3390/molecules27248997

**Published:** 2022-12-16

**Authors:** Marta Chołuj, Paweł Lipkowski, Wojciech Bartkowiak

**Affiliations:** Department of Physical and Quantum Chemistry, Wrocław University of Science and Technology, Wybrzeże Wyspiańskiego 27, 50-370 Wrocław, Poland

**Keywords:** HeH^+^, spatial confinement, harmonic oscillator potential, electric properties, dipole moment, polarizability, hyperpolarizability

## Abstract

In the present study, the influence of spatial confinement on the bond length as well as dipole moment, polarizability and (hyper)polarizabilities of HeH${}^{+}$ ion was analyzed. The effect of spatial confinement was modelled by cylindrically symmetric harmonic oscillator potential, that can be used to mimic high pressure conditions. Based on the conducted research it was found that the spatial confinement significantly affects the investigated properties. Increasing the confinement strength leads to a substantial decrease of their values. This work may be of particular interest for astrochemistry as HeH${}^{+}$ is believed to be the first compound to form in the Universe.

## 1. Introduction

The HeH${}^{+}$ is a stable, highly reactive, heteronuclear, closed shell ion. It was first discovered in the laboratory in 1925 by T. R. Hogness and E. G. Lunn at the University of California, Berkeley [1]. In the late 1970s it was suggested that it might exist in interstellar space [2,3,4,5]. Moreover, HeH${}^{+}$ is believed to be the first compound to form, through radiative association of neutral helium atoms with ionized hydrogen, after the Big Bang, when extremely hot and dense young Universe started to expand and cool down. Therefore, for many years scientists put much effort into finding HeH${}^{+}$ traces in the astrophysical plasma. Few decades of intensive search ended in failure. Finally, in 2019 R. Güsten and his coworkers published the results of the first observation of HeH${}^{+}$ in the planetary nebula NGC 7027, that was done with the far infrared spectrometer GREAT (the German Receiver for Astronomy at Terahertz Frequencies) installed on the flying observatory SOFIA (the Stratospheric Observatory for Infrared Astronomy) [6]. All that contributed to the increased interest in the studies on various physical and chemical properties of HeH${}^{+}$ [7,8,9,10,11,12,13,14,15,16].

The main goal of the present work is to analyze the impact of spatial confinement on the geometry and electric properties, i.e., dipole moment, polarizability as well as first and second hyperpolarizability, of HeH${}^{+}$ ion. In doing so, the harmonic oscillator potential of cylindrical symmetry was applied to represent the effect of spatial confinement. Note that such approach of modelling the spatial confinement can be used to mimic the confining molecular cage, especially a nanotube, or high pressure conditions. The latter one is particularly important for astrochemistry, as this branch of science investigates chemical species in space, e.g., inside stars, where they may experience extreme conditions, such as high pressure [17]. The spatial confinement phenomenon is widely recognized in the literature and has been extensively studied for many years [18,19,20,21,22,23,24,25,26,27,28]. It has been shown that the spatial confinement in the form of external repulsive potential can significantly modify properties of atoms, molecules, ions and molecular complexes [18,20,28]. Based on the research conducted thus far it was observed that the presence of such potentials causes, *inter alia*, an increase of the total energy of the considered chemical object [20,29,30], separation of the HOMO and LUMO orbitals [31,32,33], shortening of bonds [34,35] as well as substantial changes of the electric properties values [28,36,37,38,39,40,41,42,43,44,45], two-photon optical response [46] and IR frequencies and intensities [47]. The cylindrically symmetric harmonic oscillator potential is the most commonly applied in the quantum-chemical studies of the spatial restriction phenomenon. Further analysis of the impact of confining environment on atomic and molecular systems is required to broaden and systematize the knowledge on this subject.

## 2. Methodology

In the present study, the spatial confinement was modelled by the harmonic oscillator potential of cylindrical symmetry:

(1) $$V_{conf}\left({\vec{r}}_{i}\right)=\frac{1}{2}\omega^{2}\left(x_{i}^{2}+y_{i}^{2}\right),$$

where ${\vec{r}}_{i}=\left(x_{i},y_{i}\right)$ vector denotes the position of *i*-th electron. The sum of one-electron contributions, that are described by Equation (1), was added to the Hamiltonian of an isolated HeH${}^{+}$ ion (${\hat{H}}^{\left(0\right)}$):

(2) $$\hat{H}={\hat{H}}^{\left(0\right)}+\sum_{i=1}^{N}\frac{1}{2}\omega^{2}\left(x_{i}^{2}+y_{i}^{2}\right),$$

where *N* is the number of electrons. By changing the ω parameter in Equations (1) and (2) one can control the confinement strength. In order to analyze how spatial confinement of varying strength affects the bond length and electric properties of HeH${}^{+}$, the ω values between 0 and 0.8 a.u. were considered.

In all calculations the CCSD method was employed. The geometry of the isolated HeH${}^{+}$ (i.e., without the confinement) was optimized using a series of Dunning’s augmented correlation-consistent polarized basis sets. The aug-cc-pV6Z basis set was selected for the confinement-dependent optimization. The bond lengths of HeH${}^{+}$ embedded in cylindrically symmetric harmonic oscillator potential were obtained separately for each considered ω value employing the procedure developed by Luis et al. [48] The geometries of HeH${}^{+}$, optimized in spatial confinement, were used in the calculations of electric properties. The longitudinal components of dipole moment ($\mu_{z}$), polarizability ($\alpha_{zz}$), first hyperpolarizability ($\beta_{zzz}$) and second hyperpolarizability ($\gamma_{zzzz}$), reported in this work, were calculated at the CCSD/aug-cc-pV6Z level of theory using the finite-filed method, i.e., by numerical differentiation of the electronic energy with respect to the electric field. During the computations the self consistent field (SCF) convergence threshold and the CCSD energy convergence criterion were set to 10${}^{-12}$ and 10${}^{-10}$ hartree, respectively, and the pruned (99 590) grid was use. The Romberg-Rutishauser scheme [49], with the following electric field amplitudes ±0.002, ±0.004, ±0.008, ±0.016, ±0.032, ±0.064, ±0.128, ±0.256, was applied to control the numerical accuracy of the results. All computations were carried out with the aid of Gaussian 16, Revision C.01, package [50].

## 3. Results and Discussion

The first step in our study was to analyze how the choice of the basis set affects the bond length of HeH${}^{+}$ ion. In doing so, we performed the geometry optimization for the isolated (i.e., without the confinement) HeH${}^{+}$ using CCSD method and a series of Dunning’s augmented correlation consistent polarized basis sets. Then, we verified the quality of the results against high accuracy value of the bond length of HeH${}^{+}$ reported in the paper by Pachucki that is equal to R=1.463283 a.u. [12] The findings of our computations are collected in Table 1 together with the relative errors calculated with respect to the reference *R* value. Note that we were not able to optimize the HeH${}^{+}$ structure for three largest basis sets in the series, i.e., q-aug-cc-pV6Z, 5-aug-cc-pV5Z and 5-aug-cc-pV6Z. The results calculated using standard singly augmented correlation consistent basis sets (aug-cc-pVXZ, X = D,T,Q,5,6) show that the bond length of HeH${}^{+}$ becomes shorter with increasing the size of the basis set. The largest error with respect to the reference value was obtained for aug-cc-pVDZ basis set (1.6%). All other basis sets in this group gives errors far below 1%. The HeH${}^{+}$ bond length computed using the most extended aug-cc-pV6Z basis set stays in very good agreement with the reference one (error equals 0.006%). It is clear from the data presented in Table 1 that the same observations can be made for all other groups of basis sets, i.e., d-aug-cc-pVXZ, t-aug-cc-pVXZ, q-aug-cc-pVXZ and 5-aug-cc-pVXZ. The comparison of the values obtained using aug-cc-pVXZ, d-aug-cc-pVXZ, t-aug-cc-pVXZ, q-aug-cc-pVXZ and 5-aug-cc-pVXZ indicates that adding more diffuse functions to the basis set causes very small changes in the bond length of HeH${}^{+}$. Only in the case of aug-cc-pVDZ further basis set saturation leads to a noticeable decrease of bond length. The best value of HeH${}^{+}$ bond length, i.e., the closest to the reference, was achieved employing t-aug-cc-pV6Z. It is worth emphasizing that this value is only 0.00075% smaller than the one calculated with aug-cc-pV6Z. One can conclude that HeH${}^{+}$ presents low sensitivity to the basis set choice in the calculations of bond length.

**Figure 1:**
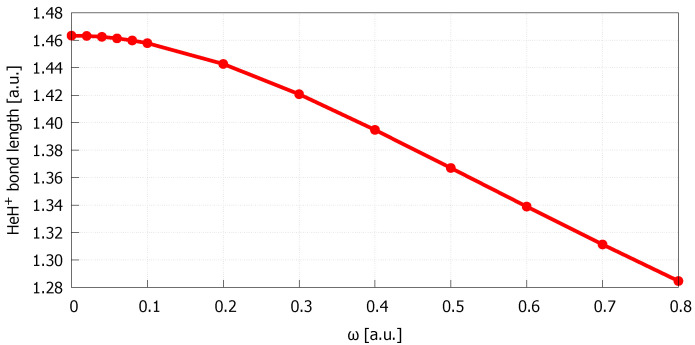
Bond lengths of HeH${}^{+}$ embedded in cylindrically symmetric harmonic oscillator potential of ω strength obtained at the CCSD/aug-cc-pV6Z level of theory.

The HeH${}^{+}$ geometry optimization procedure in the presence of cylindrically symmetric harmonic oscillator potential was carried out using CCSD method in connection with aug-cc-pV6Z, as this basis set was shown to be the smallest one, among all considered herein, that provides the bond length for the isolated HeH${}^{+}$ in close agreement with the reference. The bond lengths of HeH${}^{+}$ obtained for different confinement strengths ranging from 0 to 0.8 a.u. are collected in Table 2 and depicted in Figure 1. As can be seen, there is a quite significant decrease of the bond length values upon increasing the confinement strength. The result obtained for ω=0.8 a.u. is 12% smaller than the bond length of the isolated HeH${}^{+}$ (ω=0 a.u.). The reduction of interatomic distances, reported previously in many theoretical studies on the properties of spatially confined molecular systems [39,41,43,51,52,53], comes from changes in the distribution of electron density, which under the influence of confining potential accumulates in the spaces between atoms. Such behaviour of the electron density is a result of Pauli repulsive interactions of the spatially restricted chemical object with confining medium [34,35].

**Figure 2:**
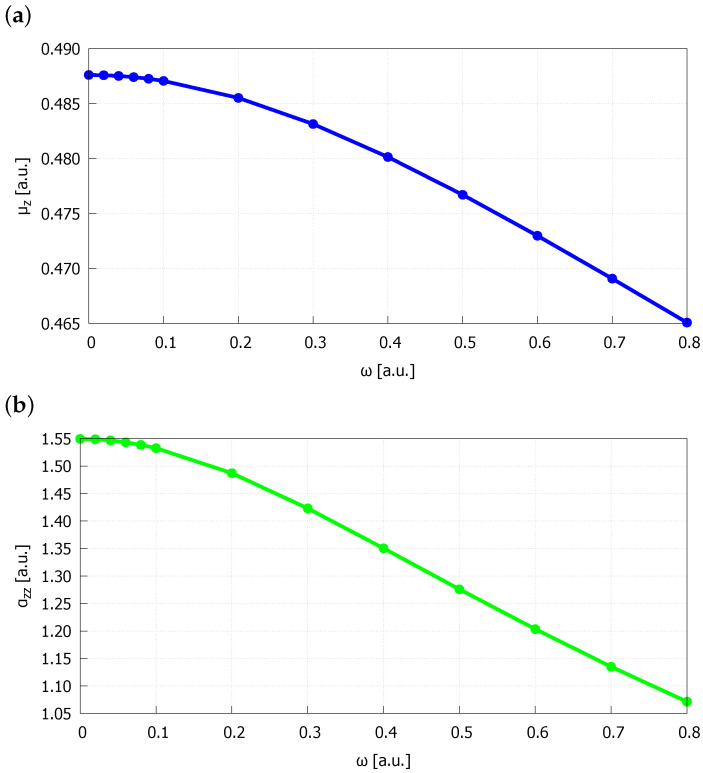

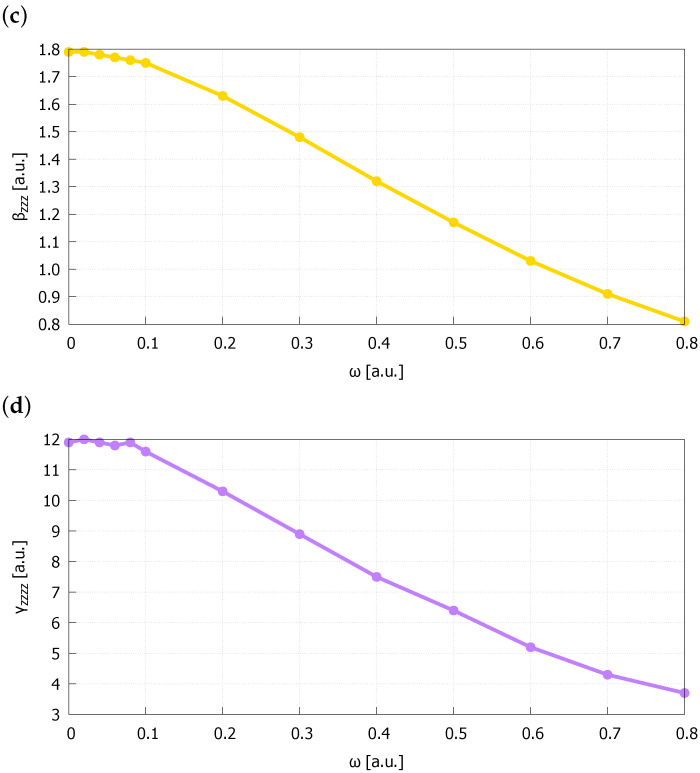
(**a**) Dipole moment ($\mu_{z}$), (**b**) polarizability ($\alpha_{zz}$), (**c**) first hyperpolarizability ($\beta_{zzz}$) and (**d**) second hyperpolarizability ($\gamma_{zzzz}$) of HeH${}^{+}$ embedded in cylindrically symmetric harmonic oscillator potential of ω strength obtained at the CCSD/aug-cc-pV6Z level of theory.

In this work, the calculations of electric properties for the spatially restricted HeH${}^{+}$ were performed for the geometries optimized in the presence of confining harmonic oscillator potential of cylindrical symmetry. The importance of the geometry optimization in spatial confinement was thoroughly discussed in many literature reports [38,41,43,52,53,54]. In real experimental situations where a chemical object is enclosed within a confining environment (i.e., subjected to the high pressure or embedded in molecular cage) the changes in electric properties will come not only from the spatial confinement effect itself, but also from the geometry distortion that appears in such conditions. Therefore, there is no doubt that the confinement-dependent geometry optimization should be considered in the studies on the spatial confinement phenomenon. The values of dipole moment ($\mu_{z}$), polarizability ($\alpha_{zz}$), first hyperpolarizability ($\beta_{zzz}$) and second hyperpolarizability ($\gamma_{zzzz}$) obtained in the present study for HeH${}^{+}$ ion under spatial confinement and without it (ω = 0 a.u.) are collected in Table 2 and illustrated in Figure 2. It is clear from these data, that increasing the pressure exerted by the harmonic oscillator potential leads to a significant decrease of all studied properties. At this stage it is worth recalling that the presence of confining potential causes a compression of electron density and consequently a reduction of molecular volume of a chemical object. Therefore, polarizability, which correlates with molecular volume, always decreases upon embedding in confining potential. Similarly, a diminishment of second hyperpolarizability under the influence of confining potential is also observed. On the other hand, the behaviour of dipole moment and first hyperpolarizability varies depending on the considered chemical system. Nevertheless, the net changes in the optical response of atoms, molecules, ions and complexes, caused by the spatial confinement in the form of harmonic oscillator potential, are always significant [28]. The results obtained in this study show that the influence of spatial confinement grows with increasing the order of electrical response, i.e., the smallest changes are observed for the dipole moment, whereas the second hyperpolarizability is altered to the largest extent. The values of dipole moment, polarizability, first hyperpolarizability and second hyperpolarizability calculated for HeH${}^{+}$ in spatial confinement of ω=0.8 a.u. strength are, respectively, 5%, 31%, 55% and 69% smaller than these obtained in vacuum (ω=0 a.u.). Note that there is a nonmonotonic behaviour of $\gamma_{zzzz}$ in the region of small confinement strengths (ω = 0–0.08 a.u.). The unexpected, although almost negligible, growths of $\gamma_{zzzz}$ are most likely a result of some small numerical instabilities in the applied differentiation procedure. Polarizability and hyperpolarizabilities of the isolated HeH${}^{+}$ (ω = 0 a.u.) were studied over the years using different methods [8,9,10,13]. The values obtained in this work are in good agreement with some of the results reported previously in the literature [9,13]. It should be emphasized that for ions dipole moment depends on the choice of the origin of the coordinate system. In this work HeH${}^{+}$ ion was oriented along z-axis, whereas hydrogen atom was positioned at the origin of Cartesian coordinate system and helium atom was placed in the positive direction of z-axis.

In summary, in the present study we analyzed the influence of spatial confinement in the form of cylindrically symmetric harmonic oscillator potential, that can be used to model high pressure conditions, on the geometry and electric properties of HeH${}^{+}$. The obtained results show that the spatial confinement significantly affects the bond length of HeH${}^{+}$ as well as its dipole moment, polarizability and (hyper)polarizabilities. Increasing the confinement strength leads to a substantial decrease of all studied properties.

## Figures and Tables

**Table 1:** The bond lengths of the isolated HeH${}^{+}$ obtained using CCSD method and various basis sets and relative errors calculated with respect to the reference bond length value (R=1.463283 a.u.).

Basis Set	Bond Length [au]	Error [%]
aug-cc-pVDZ	1.486696	1.600
aug-cc-pVTZ	1.466689	0.233
aug-cc-pVQZ	1.464062	0.053
aug-cc-pV5Z	1.463513	0.016
aug-cc-pV6Z	1.463369	0.006
d-aug-cc-pVDZ	1.486466	1.584
d-aug-cc-pVTZ	1.466693	0.233
d-aug-cc-pVQZ	1.464062	0.053
d-aug-cc-pV5Z	1.463512	0.016
d-aug-cc-pV6Z	1.463377	0.006
t-aug-cc-pVDZ	1.486432	1.582
t-aug-cc-pVTZ	1.466704	0.234
t-aug-cc-pVQZ	1.464068	0.054
t-aug-cc-pV5Z	1.463509	0.015
t-aug-cc-pV6Z	1.463358	0.005
q-aug-cc-pVDZ	1.486418	1.581
q-aug-cc-pVTZ	1.466706	0.234
q-aug-cc-pVQZ	1.464068	0.054
q-aug-cc-pV5Z	1.463512	0.016
5-aug-cc-pVDZ	1.486415	1.581
5-aug-cc-pVTZ	1.466706	0.234
5-aug-cc-pVQZ	1.464068	0.054

**Table 2:** Bond lengths, dipole moment ($\mu_{z}$), polarizability ($\alpha_{zz}$), first hyperpolarizability ($\beta_{zzz}$) and second hyperpolarizability ($\gamma_{zzzz}$) obtained at the CCSD/aug-cc-pV6Z level of theory for HeH${}^{+}$ embedded in cylindrically symmetric harmonic oscillator potential of ω strength. All values are given in a.u.

ω	Bond Length	$\mu_{z}$	$\alpha_{\mathrm{zz}}$	$\beta_{\mathrm{zzz}}$	$\gamma_{\mathrm{zzzz}}$
0.00	1.463369	0.487606	1.54943	1.79	11.9
0.02	1.463145	0.487584	1.54874	1.79	12.0
0.04	1.462483	0.487519	1.54669	1.78	11.9
0.06	1.461369	0.487411	1.54328	1.77	11.8
0.08	1.459846	0.487260	1.53859	1.76	11.9
0.10	1.457895	0.487067	1.53266	1.75	11.6
0.20	1.442737	0.485523	1.48709	1.63	10.3
0.30	1.420718	0.483147	1.42306	1.48	8.9
0.40	1.394746	0.480146	1.35040	1.32	7.5
0.50	1.367002	0.476706	1.27587	1.17	6.4
0.60	1.338911	0.472980	1.20339	1.03	5.2
0.70	1.311323	0.469080	1.13495	0.91	4.3
0.80	1.284713	0.465084	1.07136	0.81	3.7

## Data Availability

Not applicable.
